# Changes in health-related quality of life in elderly men after 12 weeks of strength training

**DOI:** 10.1186/s11556-017-0177-3

**Published:** 2017-05-30

**Authors:** Kristin Haraldstad, Gudrun Rohde, Tonje Holte Stea, Hilde Lohne-Seiler, Ken Hetlelid, Gøran Paulsen, Sveinung Berntsen

**Affiliations:** 1 Faculty of Health and Sport Sciences, University of Agder, Kristiansand, Norway; 2 Department of Physical Performance, Norwegian School of Sport Sciences, Oslo, Norway; 3 The Norwegian Olympic and Paralympic Committee and Confederation of Sport, Oslo, Norway

**Keywords:** Elderly, HRQOL, Physical activity, SF-12, Strength training

## Abstract

**Background:**

Muscular strength is associated with functional ability in elderly, and older adults are recommended to perform muscle-strengthening exercise. Understanding how improved muscle strength and -mass influence general and specific domains of quality of life is important when planning health promotion efforts targeting older adults. The aims of the present study were to describe changes in health-related quality of life (HRQOL) in older men participating in 12 weeks of systematic strength training, and to investigate whether improvements in muscle strength and muscle mass are associated with enhancements in HRQOL.

**Methods:**

We recruited 49 men aged 60–81 years to participate in an intervention study with pre-post assessment. The participants completed a 12-week strength training program consisting of three sessions per week. Tests and measurements aimed at assessing change in HRQOL, and changes in physical performance (maximal strength) and physiological characteristics. HRQOL was measured using the 12-item short-form survey (SF-12). Muscle mass was assessed based on changes in lean mass (leg, trunk, arm, and total), and strength was measured as one-repetition maximum in leg extension, leg press, and biceps curl.

**Results:**

Two of the eight HRQOL SF-12 scores, role physical and general health, and the physical component summary scores, increased significantly during the intervention period. Small significant positive correlations were identified between improvements in muscle strength, and better physical and social function. Moreover, a significant increase in total muscle mass was seen during the intervention period.

**Conclusions:**

The positive, findings from this study would suggest that systematic strength training seems to be a beneficial intervention to improve HRQOL, muscle strength and muscle mass in older men.

## Background

As aging is related to changes in mental and physical health, including loss of muscle mass and muscle function [1], an increase in the number of older adults in the total population will have a major impact on health policies and programs. A key public health goal is to improve physical fitness and health-related quality of life (HRQOL) during the aging process [2]. Regular physical activity (PA) in older adults is important for healthy aging and reduces mortality, morbidity, and injury risk [3]. PA is associated with better function in daily life, and promotes both physical and mental health, and is effective in preventing age-related loss of muscle mass and several age-related diseases [4–7].

One important aspect of PA is strength training. Earlier studies have shown that strength training is important for elderly; it is an effective way for increasing muscle strength, and have a positive effect on risk factors for age-related diseases or disabilities [5]. Muscular strength alone is independently associated with functional ability in the elderly [8], and older adults are recommended to perform muscle-strengthening exercise [9].

According to the World Health Organization, PA may play an important role in healthy aging and in promoting good quality of life [10]. HRQOL is considered a relevant indicator of subjective health and well-being, and is regarded as an important supplement to traditional biomedical health measures. HRQOL is generally conceptualized as a multidimensional construct that includes the individual’s subjective perspective on their physical, psychological, social, and functional health [11]. Self-reported health and HRQOL generate important health information for both individuals and populations, and are significant predictors of mortality, especially in older adults [12].

Most research on PA and HRQOL has focused on endurance-based exercise rather than strength training, even though the importance of strength training is underlined in the guidelines, which recommends older adults to perform exercises that increase large muscle group strength 2 or more days a week [9, 13] Moreover, few intervention studies have been conducted in this field [7, 14].

However, some studies have demonstrated the benefits of strength training on health, and in recent years, some studies have illustrated that strength training is associated with improved HRQOL in older adults [3, 15–17], and strength training has been suggested to play an important role in increasing HRQOL among older adults by improving physiological and psychological function [5, 6, 13, 18, 19]. In a systematic review focusing on PA and HRQOL, Bize et al. [16] underlined the need for more research on HRQOL and different types of PA in older adults, and in particular the association between PA and diverse domains of HRQOL. There is a specific need for more studies on strength training and HRQOL, and amount of strength training necessary to maintain or enhance strength, function, and HRQOL in older adults. Understanding the ways in which strength training influences general and specific domains of HRQOL is important for health promotion in older adults.

The aims of the present study were to describe changes in HRQOL in older men participating in 12 weeks of systematic strength training, and to investigate whether improvements in muscle strength and muscle mass are associated with enhancements in HRQOL.

## Method

### Subjects and design

The present study was an intervention study with pre-post assessment. Participants were recruited through local newspaper advertisements and invited to an information meeting and screened for study inclusion. Inclusion criteria were as follows: male, aged 60–81 years, healthy and able to participate in heavy strength training. A cardiologist conducted the medical screening prior to study entry. Exclusion criteria were as follows: any overt disease (e.g., COPD, cancer, heart disease), inability to perform resistance exercise, use of medication or supplements that could interfere with the study measures, and participating in systematic resistance exercise during the 6 months prior to study entry. Written informed consent was obtained from all participants. We recruited 70 volunteers to participate in the study, of those 19 were excluded after the medical tests, and two participants did not complete the intervention. A total of 49 men completed the intervention.

### Intervention

All subjects participated in a 12-week strength training program that had an undulating periodized profile [20], and included three full-body sessions per week. Weight load was adjusted weekly, and the volume was increased progressively throughout the 12 weeks by two well-qualified instructors who supervised the participants. The details of the strength training program have been reported previously [20, 21]. Tests and assessments were conducted before and after the training periods. The present study is a part of a larger study, where one of the aims where to investigate the effect of antioxidant supplements on muscle mass and maximal strength [20], and the participants were randomized into three groups.

However, as no significant group differences regarding changes in HRQOL were identified between the groups during the 12-week period, the groups were pooled and analyzed as a unit. The main outcome of the study was a change in HRQOL. Secondary outcomes were a change in muscle mass and muscle strength.

This study was approved by the Regional Committee (South East-Norway; S-REK: 2010/1352) for Medical and Health Research Ethics and the Data Inspectorate of Norway.

### Measures

HRQOL was measured using the 12-item short-form survey (SF-12). The SF-12 consists of 12 items measuring the following eight concepts: physical functioning; role physical due to physical problems; role emotional due to limitations in emotional health; mental health; bodily pain; general health; vitality; and social functioning, which can be combined into two sum scales, physical and mental sum scales, that reflect physical and mental health, respectively. The physical component summary and mental component summary have been scored using norm-based standards and transformed so that the general population has a mean of 50 and a standard deviation (SD) of 10 [22, 23]. For incomplete questionnaires, missing value substitution was based on the developers’ scale instructions. The SF-12 scales were scored according to published scoring procedures; each is expressed as a value from 0 to 100, where 100 represents excellent health [22].

The methods and procedures used in the present study have previously been presented in detail [20]. Prior to the intervention period, participants were given a 2-week period of familiarization with the strength training program and tests. Briefly, muscle mass was assessed by changes in lean mass (leg, trunk, arm and total) using dual-energy X-ray absorptiometry DXA(GE-Lunar Prodigy, Madison, WI, USA). Participants were scanned from head to toe in a supine position, providing values for bone mineral content, lean mass, and fat mass. Muscle strength was measured as one-repetition maximum (1RM) in the leg extension, leg press, and scott curl (elbow flexors). Each leg and arm was tested separately. After a general warm up (5 min Walking or bicycling), the loads were individually adjusted so the participants did not fatigue their muscles. We used TechnoGym exercise machine (TechnoGym, Cesena, Italy). Body mass was measured before and after the intervention [20].

### Statistical analysis

Statistical analyses were carried out using the Statistical Package for Social Sciences (SPSS) for Windows (version 22.0; IBM Corp., Armonk, NY, USA). Results are presented as means and SDs and numbers and percentages (%). Differences between pre- and posttests were analyzed using paired-sample *t*-tests for continuous variables. Effect sizes were calculated by subtracting the mean score at baseline from those at 12-week follow-up and dividing by the SD at baseline. Effect sizes, which allow for comparison across dependent variables, were interpreted according to Cohen’s effect size index [24]. Pearson’s r was used to examine correlations between changes in muscle strength and muscle mass, and the SF-12 domains [25]. The level of statistical significance was set to 5%.

## Results

### Changes in HRQOL after the 12-week strength training program

The mean age of the participants was 68.3 ± 6.1 years (range, 60–81 years).

Our results showed that two of the eight SF-12 HRQOL dimensions increased significantly during the intervention period: role physical (*p* = 0.040), and general health (*p* = 0.001). Furthermore, the physical component summary score had significant change. Physical function, role physical, bodily pain, vitality, physical component summary score, and mental component summary score showed a small\modest effect size from baseline to follow-up, while general health showed a moderate effect size (Table 1).

**Table 1:** Comparing HRQOL (mean ± SD) before and after the 12-week strength training program *N* = 49

*SF-12 HRQOL*	Mean (±SD) before	Mean (±SD) after	Effect size
Physical function	93.4 (16.0)	96.4 (10.2)	0.21
Role physical	92.9 (14.2)	96.7 (9.7)^a^	0.32
Bodily pain	89.9 (16.1)	93.4 (13.3)	0.23
General health	72.0 (17.2)	79.7 (15.6)^b^	0.54
Vitality	70.9 (21.3)	74.0 (19.7)	0.20
Social function	93.9 (14.0)	94.9 (16.1)	0.12
Role emotional	95.8 (11.2)	97.4 (8.1)	0.10
Mental health	88.8 (12.8)	89.5 (14.3)	0.13
Physical Component Summary	53.3 (5.0)	55.0 (3.3)^b^	0.32
Mental Component Summary	56.0 (5.3)	56.4 (5.2)	0.24

SF-12 range 0-100, where 100 = high HRQOL

Paired-sample *t*-test

^a,^ ^b^Indicate significant changes between baseline and 12-week follow-up; ^a^significant at 0.05 level, ^b^significant at 0.01 level

Although we treated the group as a unit, we found minor differences between the groups at baseline, the group that received the placebo supplement reported significantly lower baseline scores than the other groups on the SF-12 domains vitality (*p* = 0.030) and role emotional (*p* = 0.033), and on the mental component summary score (*p* = 0.043). However, there were no differences in the SF-12 *change scores* between groups.

### Muscle strength and HRQOL

Pearson’s r was calculated to examine the relationships among the variables. As the results in Table 2 shows, a significant positive correlation was identified between changes in HRQOL and improvements in muscle strength (leg extension) and physical function (*p* = 0.042), and between lean mass (arm) and social function (*p* = 0.021). Negative correlations were identified for leg extension and social function (*p* = 0.017), and for biceps curl and general health (*p* = 0.028).

**Table 2:** Correlation between changes in HRQOL (SF-12) and changes in muscle mass or muscle strength from before and after the 12 weeks strength training program. *N* = 49

	% Diff LegEx pre-post	% Diff LeggPres pre-post	% Diff ScotCurl pre-post	% Diff arms lean mass pre-post	% Diff legs lean mass pre-post
Diff physical function pre-post test	0.295^a^	0.034	0.038	0.088	0.054
Diff role limitation physical pre-post test	-0.026	-0.136	-0.188	-0.073	0.081
Diff bodily pain pre-post test	-0.125	-0.055	-0.100	0.032	0.281
Diff general health pre-post test	0.110	0.264	-0.314^a^	-0.083	-0.104
Diff vitality pre-post test	0.117	0.094	0.008	-0.205	0.133
Diff social function pre-post test	-0.340^a^	-0.171	-0.048	0.328^a^	-0.111
Diff role emotional limitation pre-post test	-0.092	-0.051	-0.111	-0.196	0.034
Diff mental health pre-post test	-0.045	-0.024	0.145	-0.072	-0.022
Diff Physical component summary pre-post test	0.144	0.071	-0.223	0.041	0.171
Diff Mental Component Summary pre-post test	-0.203	-0.061	0.065	-0.096	-0.051

Pearson’s *r* correlation

^a^Significant at 0.05 level

% Diff = changing % pre-post test

### Muscle mass and strength

The results show that body mass increased during the 12 weeks of systematic strength training, and, a small reduction in the fat percentage was seen. There was a significant increase in total lean mass (3%, *p* ≤ 0.001). Furthermore, lean mass in the arm, leg, and trunk increased significantly during the intervention period. The effect size from baseline to follow-up was considered large for lean mass arms, leg extension, scott curl and leg press, while total lean and lean mass in legs and trunk showed a moderate effect size (Table 3).

**Table 3:** Training variables, fatt mass and body mass given as mean and standard deviation (SD) *N* = 49

Variables	Mean (±SD) before	Mean (±SD) after	Effect size
Body mass (kg)	82.8 (12.9)	83.5 (13.1)^a^	0.05
Fat mass (%)	26.8 (6.9)	26.0 (6.5)^a^	0.11
Total lean mass (kg)	82.8 (12.6)	84.4 (6.4)^a^	0.12
Lean mass in legs (kg)	19.0 (2.4)	19.5 (2.4)^a^	0.21
Lean mass in trunk (kg)	27.7 (3.3)	28.3 (3.3)^a^	0.18
Lean mass in arms (kg)	6.9 (0.9)	7.4 (0.9)^a^	0.56
1RM in leg extension (kg)	84.5 (15.9)	99.3 (19.4)^a^	0.93
1RM in scott curl (kg)	10.5 (2.0)	12.3 (2.2)^a^	0.90
1RM in leg press (kg)	310.8 (63.9)	365.0 (81.3)^a^	0.84

*1RM* One-repetition maximum

Paired-sample *t*-test

Lean mass was assessed using dual-energy x-ray absorptiometry

^a,b^Indicate significant changes between baseline and 12-week follow-up; ^a^significant at 0.01 level

## Discussion

In the present study, an increase in HRQOL accompanied by an increase in total muscle mass and muscle strength, was seen in older adults following 12 weeks of strength training. Further, the improvement in strength was related to better physical and social function, and to a perception of better general health, although the association can be considered as modest. According to the increase in HRQOL, it is notable that, despite their baseline scores being above national norms [26], two of the eight HRQOL SF-12 scores, role physical and general health, increased, as did physical component summary scores. The SF-12 role physical domain addresses physical health-related role limitations, including limitations regarding type of work or other usual activities, and fewer accomplishments than the respondent would have liked. The general health scale reflects the respondents’ consideration of their own health [23]. Our results showed an increase in these domains after the intervention, indicating that systematic training may be positive for the participants. Strength training may contribute to a feeling that one has fewer physical limitations and can more easily carry out everyday activities such as walking long distances, climbing stairs, and balancing [27]. This may be positive for the perception of own health status. Better physical health is essential for individual autonomy, and improved muscle strength may contribute to better function and greater activity [14].

Strength training may improve not only muscle strength and mobility, but also physical and social capabilities, including older adults’ capability of performing both simple and more complex daily activities [1]. General muscular weakness is associated with aging, and even small improvements in strength and mobility can be considered important [28]. Our findings also support the notion that promoting systematic strength training in older men may have an impact beyond functional capacity, as it was associated with a positive perception of one’s own health. Another explanation for the improvement in HRQOL may be the positive social aspects of being part of a group, which may have positive psychological and physiological effects [7].

The benefits of PA on health are well known from earlier studies, but the relationship between type of PA and HRQOL have not been well described. Our findings are in accordance with Pihl et al. [19], who demonstrated that HRQOL significantly improved in older adults (mean age, 76.2 years) who were part of a strength training intervention group as measured using the SF-36; general health and the physical component scores were significantly improved at 3 months compared with the control group [19]. A study among older American adults (>65 years) showed that progressive resistance and balance training were associated with enhanced HRQOL [13], and a study among older Finnish adults demonstrated positive effects of combined strength and endurance training on some dimensions of HRQOL measured with SF-36 [5]. However, unlike our study, these studies included both older men and women.

In contrast to our findings, the results of a recent Norwegian intervention study showed no effect on self-rated health on the physical or mental subdomains of the SF-12 after 3 months of strength training in older adults recovering from hip fracture [29]. However, our study was conducted in a healthy population of older men, and one must bear this in mind when comparing these findings.

One previous study explored the relationship between different types of group exercise and HRQOL, and concluded that intense exercise and strength training had the greatest effect on HRQOL [7]. In contrast to our study, however, this latter mentioned study included both middle-aged women and men.

Findings from our study also showed a significant increase in total muscle mass and muscle strength in the arms, legs, and trunk after the 12-week strength training program. Strength training is considered important to prevent general muscular weakness, which is associated with aging [28]. The importance of strength training for older adults is also underlined in the guidelines, which recommends older adults to perform exercises that increase large muscle group strength 2 or more days a week [9].

Weak to moderate positive correlations were found between changes in leg extension and physical function, and also between arm and social function in our study. This shows that improvements in strength may be important in relation to HRQOL, especially the physical and social function. A study by Gary et al. [30] also demonstrated that better leg muscle strength was related to better physical function and increased HRQOL in older adults. However, we have no explanations for the negative correlations between leg extension and social function, and for biceps curl and general health.

### Strengths and limitations

The main strengths of the present study were the high attendance rate, the frequent meetings with highly qualified instructors, and the close follow-up. We included 2 weeks with familiarization to the strength training, and all measurements were performed by the same test leader and in the same order each time. However, some potential limitations have to be considered when interpreting the results of this study. Due to the lack of a control group, the possibility that other factors in addition to the strength training contributed to the improved HRQOOL and strength cannot be ruled out. The intervention duration was relatively short, and the sample size was small, which may have contributed to type II errors. Moreover, our results showed that baseline SF-12 scores were significantly higher than SF-36 scores for the general population in this age group [26], indicating that our sample included relatively healthy older men who may not be representative of the older Norwegian adult population. Only men were included in the study, and the results might have been different if also women were included.

## Conclusion

The findings from this study would suggest that systematic strength training seems to be a beneficial intervention to improve HRQOL, muscle strength and muscle mass among older men. HRQOL increased within role physical, general health, and physical sum scores. Moreover, modest positive correlations were found between improvements in muscle strength and better physical and social function.

From a public health perspective, the results from our study may support the notion of keeping active in order to maintain functional independence and HRQOL. One of the greatest public health challenges is to increase the number of years of healthy and high-quality life. Health professionals need to be more effective in encouraging older adults to participate in PA and strength training so that they may benefit from higher HRQOL. Further studies that include participants with lower HRQOL are warranted, and there is also a need for studies examining the long-term effects of strength training on HRQOL.

## Abbreviations

HRQOL: Health-related quality of life
PA: Physical activity
SF-12: Short form 12-item health survey
